# Adoption Strategies and Workarounds in the Evolution of Information Infrastructures Integrating Health and Social Care Providers in Scotland: Multimethod Qualitative Case Study of the Key Information Summary

**DOI:** 10.2196/94376

**Published:** 2026-10-20

**Authors:** Varun Sai, Robin Williams, Kathrin Cresswell

**Affiliations:** 1 Usher Institute University of Edinburgh Edinburgh, Scotland United Kingdom; 2 Institute for the Study of Science, Technology and Innovation University of Edinburgh Edinburgh, Scotland United Kingdom

**Keywords:** health information exchange, information systems, intersectoral collaboration, delivery of health care, integrated

## Abstract

**Background:**

Summary care records are digital technologies designed to support information sharing and care coordination across fragmented health and social care systems. However, adoption and use of these technologies can vary across diverse health and care settings, with users using workarounds and other strategies to meet their specific information needs. In Scotland, the Key Information Summary (KIS) has been in use for over 13 years, sharing key patient information from general practitioners (GPs) with other health and care providers for future care planning.

**Objective:**

Using the KIS as a case study, this study examines how various adoption strategies and workarounds have shaped the evolution of regional information infrastructures integrating health and social care in Scotland over time.

**Methods:**

This multimethod qualitative study examined the various contextual factors influencing adoption and use of the KIS across diverse health and care settings. Data collection included interviews with KIS users from health and care settings, implementers, and vendors; documentary analysis of user guides, websites, reports, and policies; and observation of multidisciplinary team meetings and technology use. Data analysis involved a hybrid approach. The technology-people-organization-macroenvironment framework was applied to code interview, documentary, and observational data, followed by an inductive analysis informed by science and technology studies theories of information infrastructures, domestication, and workarounds to examine strategies and workarounds for adapting the KIS to local contexts and evolving needs.

**Results:**

Data comprised 54 qualitative interviews, 59 documents, 2 websites, and 20 hours of observational field notes collected between April 2024 and March 2025. Findings indicate that adoption of the KIS varied across settings and over time due to misalignment between system design, evolving expectations of integrated care, and national initiatives during the COVID-19 pandemic that repurposed its use. Professional roles and associated information needs shaped differing expectations of what constituted useful information, generating tensions between users who relied on the KIS and GPs responsible for maintaining it. Formal workarounds enabled the extension of the health care–centric infrastructure into nonclinical settings to facilitate information sharing with GPs and, in some cases, supported clinician access to the KIS during system outages. Across settings, informal user-driven repair practices enabled diverse professionals to adopt and realign the KIS with its intended use.

**Conclusions:**

This study demonstrates how regional information infrastructures integrating health and care evolve incrementally as they become embedded within local routines, practices, and existing systems. Adoption was shaped by professional roles, information needs, and users’ capabilities, with formal workarounds extending infrastructures into new settings and informal, user-driven repair practices supporting ongoing use and maintenance. Policymakers and vendors must recognize that workarounds and user-driven repair practices are central to infrastructural growth and sustainability, and understanding these strategies can inform future technology development and innovation.

## Introduction

Summary care records are digital technologies designed to facilitate information exchange across organizations, supporting collaboration and cooperative working between care settings and thereby contributing to the integration of fragmented health and care services [1,2]. These records entail information that diverse health and care professionals can draw upon to inform action within their respective organizational and professional contexts [1].

Information infrastructures are sociotechnical arrangements that support information exchange and collaborative work across organizations over extended periods [3,4]. They consist of interconnected information systems, standards, organizational arrangements, and work practices that become embedded within an installed base of existing technologies and routines [3,4]. As new digital technologies, like summary care records, are introduced, they must integrate with this installed base [3]. Consequently, in complex health and social care settings, summary care records are enacted differently across organizations to accommodate local workflows, professional roles, and information needs, often creating tensions around collaboration, standards, and information use [5-7]. In different settings, digital technologies are embedded within an installed base of work practices and information sharing mechanisms, making information infrastructures “systems of systems” composed of entrenched workflows, information systems, organizational arrangements, and diverse user information needs [3,4]. In complex and variable health and social care processes, summary care records are enacted differently across settings to accommodate local workflows, systems, and professional needs, which can create tensions among stakeholders with differing expectations of collaboration, standards, and information use [5-7].

Adoption of such digital solutions can vary across different health and care settings; in some instances, often to address usability issues and the difficulty of designing information systems that support diverse roles and activities, users use workarounds, develop shortcuts to save time, or bypass tasks [8-11]. According to Alter [10], a workaround occurs when someone modifies the usual way a system is intended to be used to overcome problems, obstacles, or inefficiencies that prevent them from achieving their goals; it is primarily shaped by the system’s design, though organizational norms may also influence its use. Workarounds are practical, goal-oriented solutions that may bypass rules, standard procedures, or limitations to get work done more effectively or efficiently [10,12]. Cresswell et al [11] highlight that formal workarounds are organizationally sanctioned methods for addressing known system shortcomings, such as usability issues, whereas informal workarounds are user-initiated adaptations aimed at achieving goals with the available systems and information, for example, to complete tasks, reduce time pressures, or compensate for system defects. While these theoretical concepts acknowledge that users use workarounds in response to infrastructural breakdown [10], the relationship between infrastructural evolution and the emergence of workarounds remains largely unexamined.

Many countries around the world are pursuing a variety of strategies aimed at integrating health and care providers using digital technologies. Scotland has followed a coherent and long-term path, particularly through the establishment of Integrated Joint Boards (IJBs) to facilitate integrated and collaborative working following the Public Bodies (Joint Working) (Scotland) Act 2014 [13,14]. The Key Information Summary (KIS) in Scotland is a summary care record, used to share key patient information from general practice (GP) records with other health and care providers involved in an individual’s care [7,15].

Scotland has a nationally shared electronic health record (EHR), the Emergency Care Summary (ECS), which was introduced to make core information from GP records available across the National Health Service (NHS) [7,15]. The ECS contains demographic information, medications prescribed by the GP, allergies, and adverse reactions, and is accessible to authorized health care professionals involved in a patient’s care. Introduced in 2012, the KIS was developed as an extension of the ECS to support the recording and sharing of future care planning information across health care settings. In addition to the information available through the ECS, the KIS includes emergency contact and next-of-kin details, documented care preferences, baseline clinical and functional status, active care and escalation plans, resuscitation decisions, medication history, and a special notes section containing anticipatory and future care information.

The KIS was designed to facilitate communication between GPs and a broad range of professionals involved in community and unscheduled care, including hospital clinicians, out-of-hours (OOH) services, the Scottish Ambulance Service (SAS), community nurses, social workers, care home staff, and other professionals involved in coordinating care. GPs acted as custodians of the KIS and were the only users with administrative rights to create and update records through GP clinical systems. Information contained within the KIS was transferred electronically twice daily from GP systems to a central NHS data center [7]. Hospital clinicians, OOH services, and the SAS could access the KIS in a read-only format through their respective clinical information systems [15]. In contrast, social workers and social care providers, including care home staff, were not initially provided with digital access to the KIS during its early implementation and instead relied on indirect communication with health care professionals [7].

As an extension of the ECS, the KIS became an integral part of Scotland’s wider information infrastructure, supporting information sharing across health and social care providers [7]. Used across multiple settings and care pathways for future care planning, the KIS functions as a shared artifact that supports coordination across organizational and professional boundaries. Initially, GPs used the REDMAP (Ready, Expect, Diagnosis, Matters, Actions, and Plans) approach, together with clinical judgment, to identify patients who would benefit from a KIS, primarily those with long-term conditions and complex care needs requiring anticipatory or future care planning [15,16]. Over time, the use of the KIS expanded to additional care pathways, including dementia, frailty, end-of-life, and reablement, reflecting changing clinical priorities and service needs [7,15]. During the COVID-19 pandemic, the Scottish government further repurposed the KIS to identify and share information on individuals considered clinically vulnerable to COVID-19. This was facilitated by using the KIS, particularly the special notes section, which enabled clinicians to rapidly record and communicate relevant information across health and care settings. We have previously examined the evolution of information infrastructures for integrating health and social care providers, using the KIS as a case study to demonstrate how successive waves of infrastructural development enabled the interlinking of previously fragmented health and care systems [7]. This work showed how integration was achieved through extending existing health care–centric infrastructures into social care settings, often requiring formal workarounds in the absence of shared data standards. While previous studies have examined how infrastructures evolve through policy initiatives, technical integration, and organizational arrangements, less attention has been given to how these infrastructures are enacted after implementation, when users encounter the technology within diverse professional practices and local contexts. In particular, limited attention has been given to how users interpret and negotiate the purpose and value of shared information artifacts within situated practices of use and how workarounds and repair practices contribute to the continued evolution of digital infrastructures.

This paper draws on a broader longitudinal study examining the long-term evolution of information infrastructures supporting health and social care integration in Scotland. By examining the KIS as a shared information artifact within a broader information infrastructure, this study provides insights into how digital technologies integrating health and care providers are domesticated, maintained, and transformed through ongoing interactions between technologies, users, organizational arrangements, and wider health and care contexts. Specifically, it examines how processes of domestication, workaround, and repair contribute to the ongoing maintenance and transformation of infrastructures through interactions between technologies, users, organizational arrangements, and wider health and care contexts.

## Methods

### Study Setting

The adoption and use of the KIS vary across regions in Scotland. Lothian, a region of Scotland, serves as an exemplar of how the KIS can be used for future care planning, linking multiple health and care providers through distinct locally developed mechanisms. In Lothian, the KIS is used across multiple care pathways to share key information concerning an individual’s care needs, resuscitation status, and medication history. While most of the information in the KIS is automatically updated from the electronic records held in GP practices, the special notes section in the KIS requires periodic review and manual entry of care needs and plans by the GP. In Lothian, health and social care partnerships, specifically the Edinburgh Health and Social Care Partnership (EHSCP), use customized templates that enable community workers, social workers, and care home carers to share updates with GP practices. GP practices also share the KIS as a PDF file with social workers involved in care pathways and provide paper-based versions to carers and residents in care homes. The KIS thus functions as an artifact shared across multiple health and care providers, with different actors using it in distinct ways to address their respective information needs. Its use in Lothian offered a valuable case for examining digitally mediated collaborative work and integration efforts, in which actors from diverse professional backgrounds adopted and domesticated a standardized digital technology embedded within the information infrastructure to meet varied informational needs and support the delivery of integrated care.

### Study Design

Using the biography of artifacts and practices (BoAP) [17], and the technology-people-organization-macroenvironment technology people organization and macroenvironment (TPOM) framework [18], this multisited ethnographic study aimed to explore the adoption and contextual use of the KIS in different health and care settings. Multisited ethnography as a methodological approach allows the researcher to explore the diverse settings of technology development, adoption, and use [19]. BoAP, as a methodological tool, enabled navigation across different times and spaces, exemplified phases of technology adoption and use, and supported reflection on the choice of setting, time, and focus of enquiry [16,18]. TPOM is an evaluation framework designed to capture technological (eg, usability and performance), socio-organizational (eg, leadership and management practices over time and organizational routines), sociotechnical (eg, user satisfaction, engagement, and work processes), and macroenvironmental factors (eg, political and economic contexts and vendor management) that shape the real-time implementation, adoption, and use of digital technologies [18]. While BoAP helped examine various stages of technology adoption and use over time, providing a longitudinal view, TPOM supported contextual enquiry into how the KIS was adopted and used across different health and care settings.

### Ethical Considerations

Ethics approval for this study was granted by the Edinburgh Medical School Research Ethics Committee (EMREC). Approval for interviews with participants in Scotland was obtained on April 17, 2024 (24-EMREC-012), and approval for observations of meetings, events, and training sessions involving technology use was granted on June 17, 2024 (24-EMREC-043). The study also received approval from the NHS Lothian Quality Improvement (QI) team. Informed consent was obtained from all participants prior to their participation in interviews and observations. Participants were informed of the purpose of the study, their right to withdraw, and how their data would be used. No prior relationship was established between the researchers and participants before recruitment. The researchers did not have a role in the implementation or operational delivery of the KIS. Participant data were deidentified and anonymized in the analysis and reporting, and identifying information was removed or omitted to protect participant confidentiality. Participants were not compensated for their participation.

### Sampling

BoAP and TPOM guided the sampling strategy, enabling the identification of technology developers, users across various health and care settings, social workers from local councils, social care workers, policymakers, and academics. Recruiting participants informing various stages of technology development, adoption, and use, across various time frames and settings, enabled a longitudinal view of KIS’s adoption and factors affecting contextual use. Here, BoAP was used to identify participants with experience across different stages of KIS’s conception, adoption, and use in various time frames and settings, allowing the capture of a longitudinal view of its adoption. TPOM’s focus on technological, sociotechnical, socio-organizational, and macroenvironmental contexts guided the recruitment of diverse stakeholders. This enabled the inclusion of participants who could provide insights into factors shaping adoption and use at the macro-, meso-, and microlevels, including usability challenges, organizational and professional practices, and the influence of national policies [18].

Snowballing and purposive sampling methods were used to recruit participants for the study. Purposive sampling enabled the recruitment of participants capable of providing context-rich insights from multiple settings, based on their involvement in the development, implementation, adoption, and use of the KIS at different points in time. QI teams of NHS Lothian and Lothian Unscheduled Care Services acted as gatekeepers for the study, assisting in identifying participants from across different health and care settings. Recruitment proceeded iteratively through purposive and snowball sampling until sufficient depth and breadth of perspectives had been obtained and saturation was achieved. Gatekeepers supported purposive sampling by identifying relevant stakeholder groups, multidisciplinary team (MDT) meetings, and care settings where the KIS was used for future care planning. They facilitated access to observations of MDT meetings and training sessions involving KIS use across different health and care contexts. The sampling strategy aimed to capture variation in perspectives and experiences of KIS adoption across professional roles and organizational settings. MDTs constituted GPs, OOH staff, clinicians and specialists from hospitals, community nurses and workers, social workers from various care pathways, and care workers from social care homes.

With respect to interviews, participants were sampled to represent a range of health and social care settings, including all 4 Lothian councils. Policymakers working in the domain of health and social care were purposively identified through national policy documents to understand how the policy landscape has evolved since the KIS was first developed and implemented. These documents were identified from the NHS and Scottish government websites via Google using the search term “Key Information Summary in Lothian and Scotland.” National and international technology vendors were also purposively included to examine the factors influencing technological development, implementation, and adoption, as well as to gather insights into user support provided over time. In addition, a snowball sampling approach was used, whereby participants were invited to suggest others involved in the development, implementation, adoption, or use of the KIS.

### Researcher Characteristics

VS (male) is trained as an IT engineer, with research experience in medical informatics, health and social care information systems, and qualitative research. KC (female) and RW (male) have research experience evaluating large-scale digital transformation initiatives across the NHS, including the Global Digital Exemplar program, which supported digital transformation across acute hospital settings in England, and NHS AI Lab programs.

### Data Collection

Data collection was undertaken by VS between April 2024 and March 2025 under the supervision of KC and RW, using multiple qualitative methods, including semistructured interviews, ethnographic observations of KIS use in practice, and documentary analysis of materials related to KIS implementation, training, and operational processes. These methods were integrated within a qualitative case study design to develop an in-depth understanding of the technological, sociotechnical, socio-organizational, and macroenvironmental factors shaping the adoption and use of the KIS within its real-world context.

One-to-one, semistructured interviews were carried out with a range of stakeholders, including users, policy leads, data scientists, academics, technology developers, and health and social care providers, to explore their perspectives on the development, implementation, adoption, and use of the KIS. Interviews lasted between 40 and 60 minutes, were conducted online via Microsoft Teams, audio-recorded, and transcribed verbatim. For a small number of participants, follow-up interviews were conducted, where participants were willing to engage further, and additional discussion was needed during the analysis. These conversations were used to clarify points raised in the initial interviews and to explore emerging questions from the ongoing analysis.

Topic guides for the interviews, informed by the TPOM framework [18], explored how system usability and performance, leadership and governance practices, organizational routines, user engagement and everyday work practices, and wider political-economic and vendor-related conditions shaped the adoption and domestication of KIS across health and social care settings. Tailored topic guides were developed for users from different settings, vendors, academics, and policy leads to capture insights regarding their role in the conception, adoption, and use of the KIS (Multimedia Appendix 1).

Nonparticipant observations were undertaken to examine how MDTs and national planning groups engaged with the KIS to support future care planning. Observations included MDT meetings involving NHS Lothian and the EHSCP, as well as national planning group meetings, to explore interprofessional collaboration across health and care organizations at both regional and national levels. Use of the KIS by different users was also observed to understand how its adoption and use varied across settings. Observation sessions lasted between 1 and 2 hours. Meetings and patterns of technology use were observed online via Microsoft Teams. In addition, in-person training sessions delivered by the EHSCP in care homes were also observed. Field notes were systematically documented using a structured observation protocol (Multimedia Appendix 2).

Study participants were also asked to provide relevant documents regarding the implementation, adoption, and use of the KIS. Internal case studies, user guides for different professionals, contingency plans during technology breakdown, and software update notifications were shared by participants. Documents were purposively selected based on their relevance to understanding the evolution of the KIS as an information infrastructure. Selection focused on materials describing the development, implementation, and use of the KIS in Scotland and Lothian; health and social care integration initiatives; the intended functions and uses of the KIS; related health and social care information systems supporting KIS access; and implementation strategies and anticipated benefits at local and national levels (Multimedia Appendix 3). Historical documents were treated as empirical artifacts that provided insights into how standards, practices, and infrastructural arrangements were developed, negotiated, and embedded over time. These documents were examined qualitatively to understand challenges surrounding the adoption and use of KIS in different settings over time and to explore the similarities and differences between national goals, technological claims, and regional narratives.

### Data Analysis

NVivo (version 14; Lumivero) was used to code the interview transcripts, observation notes, and documentary materials. Data analysis was conducted by VS under the supervision of KC and RW. VS was the primary and sole data coder, while KC and RW provided ongoing oversight of the analytical process and contributed to discussions of coding, emerging themes, and interpretations. The analysis adopted an interpretive approach that recognized the researcher’s positionality and subjectivity. Credibility was strengthened through the search for disconfirming evidence and the use of data triangulation. Triangulation involved integrating insights from all qualitative data sources to situate the case study within the broader context of integrating health and social care through digitalization while also capturing the microlevel practices of individual users and the mesolevel organizational processes and interactions that shaped the adoption and use of the KIS.

Data analysis followed a hybrid approach, that is, a combination of inductive and deductive approaches [20]. Deductive thematic analysis, informed by the TPOM framework, was applied to interviews, documents, and observational notes to examine the challenges around technology usability, user and organizational practices, as well as the wider macroenvironmental factors influencing the adoption and use of the KIS across different settings [18]. Related codes that emerged were clustered into themes and compared across sites, professional roles, and time periods to identify relevant subthemes following an inductive analysis. Concepts and theories from science and technology studies, including information infrastructures [3-6,21-23] and domestication [24,25], alongside theories and concepts on workarounds [10-12], were used to further interpret how the KIS was adopted across different settings over time as the regional information infrastructure evolved. These approaches enabled a systematic analysis of KIS’s adoption and use over time, the tensions emerging during its adoption across diverse settings, and the various adoption strategies and workarounds enacted by users and organizations to mitigate constraints imposed by the system. The coding process involved organizing codes into second-order and aggregate themes, with the resulting coding structure provided in Multimedia Appendix 4.

### Reporting Guidelines

This study was reported in accordance with the COREQ (Consolidated Criteria for Reporting Qualitative Research) guidelines [26]. The completed COREQ checklist is provided in Multimedia Appendix 5.

## Results

### Overview

Data consisted of 54 interviews; notes from observing technology use, MDT meetings, and national-level meetings regarding the use of KIS for future care planning (20 hours); and documentary analysis of 59 documents and 2 archived websites (Table 1).

**Table 1:** Dataset.

Method, dataset, and description				Values, n
**Interviews**				
	**54 interviews (lasting 40-60 minutes each)**			
		GPs^a^ from daytime practices	14	
		Social workers and social care workers	13	
		Out-of-hours service	5	
		Data analysts from Public Health Scotland	4	
		Clinical informatics and population health experts	4	
		Technology vendors and software developers	4	
		Academics and implementation scientists	4	
		Government representatives for digital health and social care	4	
		GP administrative staff	2	
**Observations**				
	**12 meetings (1-2 hours each) lasting 20 hours in total**			
		Observing national-level collaborative meetings to use digital technologies for future care planning from May 2024 to December 2024	8 hours	
		Observing events hosted by social care organizations	8 hours	
		Observing technology use by clinicians	4 hours	
**Documents**				
	**59 documents and 2 websites**			
		Meeting notes from care home meeting with GP practices for service improvement of future care planning	12	
		The KIS^b^ user guide for GPs, nurses, social workers, and social care workers	11	
		Information leaflets issued by various departments promoting the KIS	8	
		User guides and contingency plan for information systems used in different health and care settings	8	
		National and regional information sharing guidelines, regulations, and policies	5	
		Economic impact measurement plans and assessments	4	
		Documents pertaining to 7 steps to ACP^c^ for care home staff	4	
		Digital health and care strategies in Scotland	3	
		Internal case studies were around the introduction of the KIS in care homes in the northeast of Edinburgh	2	
		Internal case studies about the use of the KIS by Alzheimer Scotland Post Diagnostic Dementia Service in Edinburgh and by Reablement and End of Life Care in the northwest of Edinburgh	2	

^a^GP: general practitioner.

^b^KIS: Key Information Summary.

^c^ACP: anticipatory care planning.

The KIS was designed to enable the sharing of key patient information from GP with other service providers to support continuity of care and provide an overview of patients’ health status and care plans. However, expectations regarding the content and level of detail required varied across professional groups and care settings, and the functionality and design of KIS did not accommodate the full range of tasks it was expected to support. Consequently, tensions emerged between different user groups (such as OOH and hospital staff) and those responsible for maintaining and governing the information recorded in the KIS (GPs). These tensions were exemplified following top-down policy initiatives by the government that repurposed the KIS to identify vulnerable patient groups during the COVID-19 pandemic. As a result, many KISs were created without substantive information on future care plans and were instead flagged as having been generated as part of COVID-19 protocols. In turn, formal workarounds were used by health boards to provide social workers and carers access to the KIS and to ensure that relevant and updated information regarding future care is shared with the GP practices. Informal user-driven repair strategies were used in different health and care settings to update the KIS with relevant information and realign the KIS with its intended use. Here, repair work was conceptualized as the ongoing, often invisible labor through which users maintain, adapt, and render technologies usable in practice. Figure 1 provides an overview of identified themes.

The analysis identified several interconnected challenges that influenced the adoption, use, and ongoing maintenance of the KIS across health and social care settings. These challenges related to information quality, interoperability, professional responsibilities, and organizational capacity. Rather than representing discrete implementation failures, these challenges reflected tensions that emerged as the KIS evolved from a health care–focused summary record into an information artifact supporting wider health and social care integration. Users and organizations responded to these challenges through formal workarounds and informal repair practices that sought to maintain information quality, extend access, and align the KIS with changing care needs.

**Figure 1:**
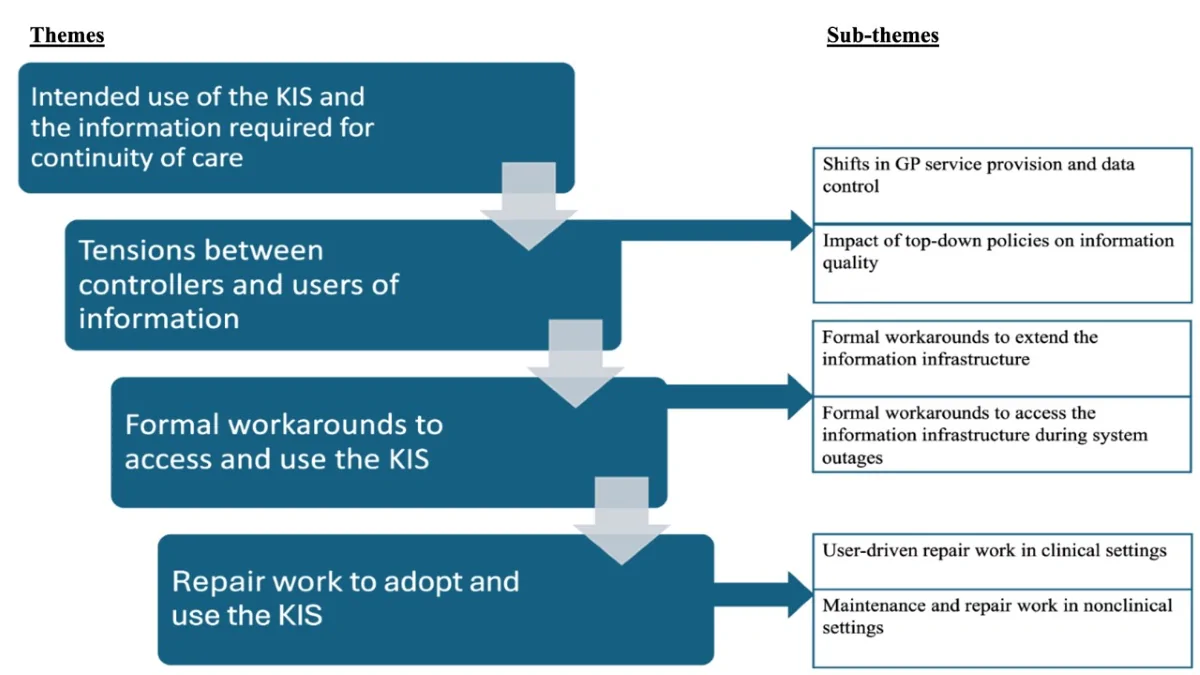
Themes emerging from the analysis. GP: general practitioner; KIS: Key Information Summary.

### Intended Use of the KIS and the Information Required for Continuity of Care

GPs were regarded as the main point of care for residents in Scotland, and the KIS was developed to share key information regarding a person’s future care plan to other primary, secondary, and community care providers. GPs identified individuals who would benefit from a KIS within their practice and documented their care plans and preferences. Consequently, the KIS enabled GPs to share information concerning a person’s medication, resuscitation status, and care plans with other health and care providers, who may not have had regular or long-term contact with the individual. Clinicians in OOH settings, who served a broader patient population than GPs and provided care outside regular GP hours, reported that the KIS gave them access to information they would not otherwise have, allowing them to understand the GP’s assessment of a patient’s condition and to make informed clinical decisions:

In an out-of-hours environment, you don’t know the patient; all I’m seeing is their KIS, and only if the GP has uploaded one. If you work in Edinburgh out of hours, you’re covering most of the city of Edinburgh. The information that the KIS summary can provide is really valuable because it helps to just get a sense of what the GPs are doing, what they’re worried about regarding a person, and what their care needs are. Those things are sort of there to help you make those decisions when it’s an emergency, night-time or evening.

Clinician, OOH (1), Lothian

The KIS was developed to support GPs in engaging with individuals about their future care with primary, secondary, and community care providers. However, as the KIS was adopted across different care settings, expectations regarding the type, completeness, and level of detail required varied between professional groups. While GPs viewed the KIS primarily as a mechanism for documenting future care preferences, clinicians in other settings increasingly relied on it as a broader source of information to support decision-making. Conversations regarding future care between the GP and the individual were regarded as an ongoing, dynamic, and iterative process aimed at capturing an individual’s care preferences, as their conditions and care needs changed. GPs, acting as custodians of KIS data, were responsible for updating patients’ care preferences and future care needs, making the KIS a dynamic record, whose completeness and accuracy varied according to the extent to which individual GPs maintained it. However, this model of data stewardship also created a key challenge, as maintaining the accuracy and completeness of KIS information depended heavily on the capacity, time, and willingness of individual GP practices.

The KIS is a living document. To me, once a KIS is in place, it should be a dialogue to know when you’ve got a new condition or if something new happens. Lives are fluid, and there will be new bits of information, and it should be an ongoing document.

Clinician, GP (3), Lothian

However, some GPs noted that they did not always maintain such long-term engagement with patients whose health and care needs had become complex, requiring them to gather relevant information from other sources to update the KIS. This variability represented an important challenge, as the usefulness of the KIS depended on sustained processes of information maintenance that were influenced by individual practice capacity, workload, and access to information from other care providers. In some cases, individuals required long-term care that could not be delivered in community settings and instead depended on ongoing support from hospitals, nursing homes, or care homes. To ensure the KIS reflected up-to-date information, GPs had to draw on multiple information sources, including updates provided by other health care professionals, social workers, and carers. For instance, discharge letters were provided by hospital-based clinicians to GPs after an individual’s discharge following hospital admission, which the GPs had to review to update the KIS. However, not all GPs adopted a systematic approach to reviewing documentation on patients’ future care needs, and maintaining these records required ongoing effort. Access to up-to-date information was particularly valuable for clinicians in hospital and OOH settings.

So we’ve got 150 to 200 housebound patients in my practice, quite high. So, we did a project around making sure all housebound patients had a KIS, and you see that sometimes you haven’t seen the patient all year, or they’ve just been seen in secondary care. But no one has thought about updating it from the secondary care letters.

Clinician, GP (6), Lothian

### Tensions Between Controllers and Users of Information

#### Overview

The variations in how GPs and GP practices updated the KIS differences reflected the evolving organization of general practice over time, resulting in uneven distributions of resources and capacity across practices. While GPs bore primary responsibility for ensuring that KIS information remained up-to-date and clinically relevant, other users largely benefited from the availability of this information, thereby generating tensions between different user groups. The demand for up-to-date information was further intensified by national initiatives that repurposed the KIS to identify vulnerable patient groups. The following sections examine these issues in detail.

#### Shifts in GP Service Provision and Data Control

The organization of GP services in Lothian was such that not all GP practices had the responsibility of providing medical services to care homes, nor were they equally resourced or willing to update the KIS. In some cases, GPs expressed the need to have control over the data collected and shared using the KIS and had the resources to ensure this. Whereas in other practices, they had no spare capacity to review and periodically update the KIS, leading to variability in the way the notes were recorded and updated.

All the onus is on the GP practice to add the information into the KIS, so it’s very much dependent on the capacity and availability of staff who are allocated that role to update the KIS. It’s different across each GP practice. You’ll have a GP practice that will very much want to keep control and be responsible for updating the KIS. Not all GP practices cover care homes; if they do, then they’ll go out, do the visit, and quite often they’ll keep that to themselves. They don’t want to share it out, not quite gatekeeping, but there is that element of them wanting to kind of have some oversight of that responsibility and the cheques.

Social work, EHSCP, Lothian

It depends on how pressurized the practices are and the amount of capacity they have. I think if a practice has no spare capacity, then people don’t dedicate the necessary time to update the KIS. Whereas another practice or another GP who has got a bit of capacity will actually spend time updating the information to make sure it’s current, so there’s a huge variability, which has been one of the frustrations.

Clinician, Hospital (1), Lothian

The introduction of the General Medical Services (GMS) contract in 2018 led to the establishment of MDTs intended to reduce pressures on GP practices and to provide integrated care. The GMS contract of 2018 also placed GPs as controllers of patient data for integrated care services. Since the earlier GMS contract of 2004, GP practices have not operated as NHS-managed services but as independent businesses contracted to deliver services on behalf of the health board. Before this restructuring, many practices were directly funded and managed by health boards; under the GMS contract of 2004, GPs functioned as independent contractors, and a pay-for-performance scheme was introduced. As a part of the GMS contract of 2018, practices that are geographically close formed clusters to work together with regional health and social care partnerships to provide integrated care. Although the 2018 contract sought to alleviate workload by positioning GPs as clinical experts supported by wider MDTs, documentary evidence and participant accounts indicated that, in practice, these changes often increased rather than reduced the workload placed on GP practices. Some of the GP practices were struggling to recruit new GPs, whereas some had the resources to recruit more staff and run integrated care services through MDTs. However, a few GPs noted that insufficient systemic and organizational support for running GP practices adversely affected their capacity to maintain high-quality and up-to-date data in the KIS. Together, these findings demonstrate how organizational capacity, workforce constraints, and variation between GP practices affected the consistent embedding and maintenance of KIS-related activities across settings. These challenges were not simply related to user willingness to adopt the KIS but reflected broader organizational conditions that shaped the capacity of practices to sustain information-sharing activities over time. While there was increased recognition of the need to provide up-to-date information for population health management and preventative care among GPs, they also questioned the misalignment of national-level priorities regarding care provisioning and delivery.

#### Impact of Top-Down Policies on Information Quality

The need for reliable and up-to-date patient information became increasingly critical after the KIS was repurposed during the COVID-19 pandemic to identify vulnerable groups of patients by the government. This was achieved by using the special notes section in the KIS to indicate that the record had been created in response to the pandemic. However, this “top-down” approach diverged from the KIS’s primary purpose as a tool to record and share an individual’s care preferences and needs. This affected the usefulness of the information available to professionals using the KIS for clinical decision-making, particularly in settings where clinicians relied on shared information to understand an individual’s current health status and care requirements. Thus, a key challenge emerged when national policy priorities expanded the expected function of the KIS without corresponding changes to the information structure, workflows, or responsibilities associated with maintaining the record. One clinician in the OOH service highlighted how the repurposing of the KIS during the pandemic affected the information available through the system:

There was a mandate during the pandemic that every patient should have a KIS summary. But the pragmatic reality was that much of what was included was focused on COVID status. It often did not capture other medical problems they might have, such as diabetes, involvement with mental health services, or multiple medications. So, it was quite patchy in terms of what up-to-date information was actually coming through to the clinician looking at the case through the ECS.

Clinician, OOH (1), Lothian

The consequences of this policy-driven repurposing were therefore not limited to information quality but also generated additional maintenance work for users attempting to restore the original purpose of the KIS. Consequently, GPs were required to undertake additional work to identify individuals who would benefit from a KIS containing up-to-date information relating to future care planning and to restore the intended purpose of the record. This illustrates how repurposing an existing information infrastructure for new policy objectives can unintentionally reshape information practices, alter the content generated within the infrastructure, and create additional maintenance work for users responsible for sustaining its intended function.

While users in OOH services and the ambulance service benefited from having up-to-date information in the KIS, maintaining this accuracy required GP practices to collate information from multiple sources and manually enter updates. Clinicians in OOH settings shared that recent and relevant information in KIS aided in decision-making and provided an improved understanding of the individual’s condition and care plans in place. Accordingly, clinicians from OOH services and hospitals emphasized the importance of involving social workers and social care workers from MDTs (such as the Care at Home, End of Life care, Post-Diagnostic Dementia Service, and Reablement teams) in updating the KIS, both to reduce the burden on GP practices and to support a more integrated approach to care.

I think sometimes the frustration is that updating the KIS feels like it is a GP’s job and it’s quite time-consuming. You have to click a lot of buttons, copy the information, and make sure it is shared. Sometimes, GPs can feel like we’re doing all this work to help other services more than to help ourselves. I don’t think that is true because it helps with continuity, and if different doctors were seeing a patient, then they would have a better understanding. It would be great if more people could get involved with creating and updating the KIS, for example, the re- enablement team or the care home teams, making it a collective joint effort rather than it always coming back to daytime GPs to do.

Clinician, OOH (3), Lothian

In some instances, when clinicians in OOH settings identified that a patient could benefit from a KIS, they would email the GP with relevant information extracted from OOH-based information systems. Although OOH staff found this process time-consuming—requiring them to gather information from multiple systems—they often did not receive any feedback from GPs regarding updates to the KIS.

There’s a bit of work involved because you’ve got to obviously do the e-mail, fill in the paperwork and the name, address the pile, put all that we have been doing, and any other information that we might have on our system about them. So, to update it is a wee bit time-consuming, but we never ever get a response back to see that it’s updated.

Clinician, OOH (2), Lothian

### Formal Workarounds to Access and Use the KIS

#### Overview

In response to challenges related to information access, interoperability, and information quality, organizations and information system vendors developed formal workarounds to extend and sustain the use of the KIS. These were designed both to extend access to KISs to nonclinical settings and to ensure continued access to the KIS during system outages. The following sections examine these subthemes in detail.

#### Formal Workarounds to Extend the Information Infrastructure

Understanding the need to update relevant information and to provide integrated care, a few GP practices in Lothian involved social workers and social care workers from MDTs and care pathways to update the KIS. However, as the information systems used by social workers and care providers were not interoperable with the information systems used by GPs, formal workarounds were used to provide social workers and care providers access to the KIS. These workarounds were formal insofar, as they were agreed and promoted by various stakeholders like the health board, technology vendor, and the EHSCP. Formal workarounds included creating proxy access for social workers and care providers involved in MDTs (Reablement, End-of-Life Care, and Post-Diagnostic Dementia Services) to view a read-only version of the KIS through hospital information systems on behalf of the NHS. Functionally, this constituted a semilegal extension of the technology, permitted through formal organizational and vendor sanctions. The boundary between formal and informal use was blurred, as access was generally limited to viewing the KIS rather than updating it, thereby avoiding potential breaches of professional jurisdiction or liability. Here, communities of practice, such as the regional health and social care partnership (EHSCP), organized training sessions for social care workers in care homes and for social workers involved in MDTs and care pathways. These training sessions helped in building a sense of ownership among nonclinical users (social workers and social care workers). EHSCP created templates for social workers and care providers to understand who would benefit from a KIS, as well as to share updates regarding future care plans with the GP practices.

Formal workarounds in providing social care workers from MDTs access to the information infrastructure also involved issuing NHS email accounts to a limited number of social care providers operating care homes. Care home managers, due to their involvement in MDTs and seniority, were provided with NHS email accounts on behalf of the NHS. This arrangement provided nonclinical entities, with access to the secure emailing system used by health care providers to share updates with GPs. Documentary evidence from NHS Lothian demonstrated that this approach was formally supported, with care homes encouraged to establish secure email connections with NHS services to facilitate the exchange of person-identifiable and sensitive information. Observations of MDT discussions and training sessions, alongside documentary evidence from EHSCP materials, demonstrated that standardized templates were developed to support care providers in identifying individuals who may benefit from a KIS and communicating relevant updates to GP practices. However, because access was limited to selected individuals within care homes, managers often acted as intermediaries, sharing information with GP practices on behalf of other members of the care team who did not have direct access to the information infrastructure.

#### Formal Workarounds to Access the Information Infrastructure During System Outages

Formal workarounds were also used and promoted by health boards and vendors when technical barriers impeded access to the KIS through various information systems used by the different users. Contingency plans were developed and shared, detailing alternative communication channels that clinicians could use to maintain continuity of care by accessing relevant information. For instance, during system outages that made the KIS temporarily unavailable, clinicians were able to obtain relevant information through telephone referrals or secure email by contacting designated support teams within the health board. These teams, such as the Flow center (a digital navigation center for assisting clinical users), coordinated the distribution of information as well as contingency plans to GP practices, OOH services, and hospital-based clinicians, ensuring continued information access during planned and unplanned system downtime.

Similarly, unanticipated impact or issues demanded the use of formal workarounds to provide temporary access to information systems to ensure continuity of care. For instance, after Adastra had a security outage in 2022, users in OOH settings had to learn and start using TrakCare (Scotland’s hospital-based information system) until Adastra was restored. Formal workarounds involved the health board and the vendor of TrakCare agreeing to provide OOH entity access to information systems used in hospitals. As part of the workaround to provide access, the OOH service was listed as a new hospital to be identified as a trusted entity with administrative rights. These workarounds ensured that staff from OOH settings could share clinical information with other entities like GPs, as well as view the KIS. However, OOH staff were required to familiarize themselves with TrakCare, a system designed for hospital-based workflows. This imposed additional learning demands and disrupted established clinical workflows for clinicians unfamiliar with hospital-based information systems.

When we had that ransomware attack, they had to quickly get everyone a proper TrakCare account so that they could write up and share the case notes. That was a huge piece of work, and they had to set up OOH as a clinic, like an outpatient clinic. So, we could at least write the GP a letter, and we could access the KIS. TrakCare wasn’t a bad option; there weren’t any problems with it necessarily. It’s just the fact that everyone had to learn it, because most of us hadn’t been working in hospitals particularly, and we would look stuff up, but we wouldn’t have to interact with the system, particularly often.

Clinician, OOH (2), Lothian

Vendors provided users with documents promoting temporary workarounds to ensure interoperability across information systems used to access the KIS. Clinicians routinely relied on multiple interconnected systems, meaning that updates to any one software created misalignments with established workflows. In response to such issues, vendors issued detailed user guides describing interim procedures to support continued use. For example, Vision, the information system used by GPs, and the Scottish Care Information (SCI) Gateway, a national platform for clinical referrals, were software applications that allow GPs to access and share information with other clinicians. A security update to the SCI Gateway restricted users to a single active session and prevented simultaneous access through multiple browsers or devices. For Vision users, however, each patient consultation triggered a new login instance (parallel active session), causing active SCI Gateway sessions to be terminated automatically. To manage this incompatibility, vendors provided Vision users with revised login procedures intended to preserve interoperability with the updated SCI Gateway. These procedures required GPs to log in and out of the system after each consultation, thereby inadvertently disrupting routine practice.

### Repair Work to Adopt and Use the KIS

#### Overview

Following the COVID-19 pandemic, maintaining adoption of the KIS required users to undertake additional repair work to address challenges related to information quality, relevance, and interoperability. First, to restore information quality in the KIS, users adopted new strategies that functioned as repair work to maintain information quality. Across settings, users engaged in informal repair practices to obtain relevant information from alternative sources and to update the KIS. These practices varied by site and were influenced by users’ roles, digital skills, and access to specific information systems. Second, adoption also varied across health and social care settings. In nonclinical settings, carers and social workers acted as human nodes for information gathering and sharing, as the information systems used were not interoperable. The following sections illustrate how professionals in different settings adopted different strategies, including repair work, to realign the KIS with its intended purpose and update its information.

#### User-Driven Repair Work in Clinical Settings

As the quality of information in the KIS declined after COVID-19, users had to use different mechanisms to improve the quality of information in the KIS. In GP settings, these repair strategies were developed by GPs with analytical expertise and were shared informally with colleagues. In some GP practices, GPs with access to the Scottish Patients at Risk of Readmission and Admission (SPARRA) dataset used risk scores to identify patients who might benefit from a KIS and to determine whether existing KISs required updating. The SPARRA dataset identified individuals at risk of hospital admission by assigning a risk score derived from analyzing an individual’s chronic health conditions, prior unplanned hospital admissions, mental health diagnoses, and alcohol or drug-related admissions. However, a few GPs highlighted that reliance on SPARRA data was restrictive, as it drew predominantly on hospital-based data. Additionally, the high prevalence of multimorbidity within the dataset complicated efforts to determine which individuals would meaningfully benefit from a KIS.

The solution I’m finding at my practice is that I’m trying to use the SPARRA data, and I’m trying to get just my work through it. Okay, now who has just got the COVID phrase and who’s got a meaningful KIS? I get the SPARRA data, and then I build a group and code all of those. So, you could look at who’s coded for high SPARRA data and has a KIS and who doesn’t have a KIS and identify them. But that is really difficult now because everyone’s got cancer, even if there’s nothing in that, that’s quite difficult.

Clinician, GP (2), Lothian

SPARRA is not unhelpful, but if my recollection is right, it’s skewed by hospital admission. So it’s quite good at telling you who’s currently sick. Which is not quite the same as predicting who might become sick, and you might have something you could do about it as a GP.

Clinician, GP (5), Lothian

A few other GP practices, which were involved in MDTs tackling issues around frailty and multimorbidity, used local datasets to identify who would benefit from a KIS. These GP practices, forming a cluster under the GMS contract of 2018, ran special services to identify frail patients in a region and support their care plans and preferences. GP practices used local datasets to identify frail patients and to identify if they had a KIS in place. These informal repair strategies, developed and used in different practices, were later shared by communities of practice like Scottish Clinical Information Management in Primary Care and EHSCP. As a result, networks of GP practices, in different clusters, had started to use SPARRA datasets, alongside other local datasets capturing details regarding polypharmacy, multimorbidity, and frailty.

Clinicians working in OOH settings reported that many other sources of information could help GPs understand an individual’s current care needs, but these were stored in various formats across different information systems. This information included records from community nurses and care home staff, maintained across multiple digital systems as well as physical documents, to capture a person’s health and care needs. OOH clinicians noted that nurses and care home staff, who knew the patient and their family well, provided valuable information about next of kin, recent hospitalizations, current functional status, and any gradual health decline over time. Nurses and carers visiting house-bound individuals documented residents’ mobility, recent hospital visits or admissions, and care plans using disparate information systems—and sometimes physical copies. Because information systems across organizations were not interoperable, OOH clinicians visiting care home residents often relied on verbal reports from nurses as well as carers and shared them with GP practices via secure email, with the extent of sharing and GPs’ incorporation of updates both depending on individual capacity and willingness.

So if it’s district nurses in hospital-based complex care settings or home care for house-bound patients, it would usually be verbal communication. You would take a handover from the nurse or carer who contacted our service to know what they’re concerned about, and usually, they know the patient well and know the family, which can be an excellent source of information. Like, who is the patient’s next of kin, or say that the person has been in the hospital for a problem last month. I think the care home staff are a really valuable source of information because they can tell you about the level of function of the person, whether they have been sick for a while, or are normally quite mobile and independent but currently bed-bound or needs help, if this patient’s been on a downward trajectory over the last six months and they’ve been declining steadily. That is really useful information, and I would type up my notes and share them with the registered GP afterwards.

Clinician, OOH (2), Lothian

#### Maintenance and Repair Work in Nonclinical Settings

While formal workarounds enabled social care providers to access the secure emailing networks to receive a digital copy of the KIS from GP practices, care providers also maintained a physical copy. A few care managers highlighted that in social care settings, physical copies of care plans and the KIS were stored to ensure continuity of care and as a contingency mechanism. These contingency mechanisms were in place, recognizing the vulnerability of digital technologies to occasional breakdown, and due to epistemic practices around record keeping in care settings. The different material forms of the KIS also served as a medium to share information with clinical staff providing emergency care, like the ambulance service. Ambulance services visiting care homes often relied on multiple sources of information present at care settings while attending a patient, which required care staff to maintain physical records as well.

Yeah. I think it is good practice to have everything on the electronic platform, but like any electronic device, as you do rely on the internet and electricity working, and you know if all that went down, you are left with nothing, which I don’t feel is really good practice. This resident has to be cared for regardless of whether we have electricity, Wi-Fi, or Internet. So, we have a folder for each person that contains these very important documents. If anything were to happen to this system, or let’s say you know, like the NHS system that has been hacked several times, you don’t know what will happen in life. But I still have to provide the most important care to my resident. So, I feel we should have a paper copy just in case things happen, and we can’t access it digitally.

Social care (3), Lothian

A few GPs reported that extending the health care–centric information infrastructure to social workers and care providers to access and share information regarding future care planning was beneficial. Although this access to the information infrastructure enabled a few care workers and social workers to share information with clinicians, they functioned as human nodes who shared information with the GP practices to update the KIS. For instance, charity and voluntary organizations engaged in frailty assessments had to manually collect information regarding a person’s contact information, care plans in place, mobility status, and available resources at their residence to support home-based care. Voluntary organizations then had to share the assessment with GPs, where GPs or GP admin staff would enter the details into the KIS.

Internal implementation reports by EHSCP also highlighted the development of local forms and documentation processes across different reablement pathways to facilitate information collection and sharing between community organizations and GP practices. However, despite these efforts to support information exchange, challenges remained as different professional groups operated with distinct epistemic approaches to documentation, information recording, and care practices. These differences influenced how information was generated, interpreted, and incorporated into the KIS, requiring ongoing negotiation and coordination among stakeholders.

There are some local initiatives, like the long-term conditions team, where community teams will collect the data in a structured way and then email it to the GP practice so that it’s kind of easier for the GP. You can just copy and paste aspects of that record to share in the KIS as opposed to having to, you know, originate it and type it up yourself.

Clinician, GP (2), Lothian

## Discussion

### Summary of Findings

Findings illustrate how the KIS, as a shared object of collaboration across diverse health and care professionals within an information infrastructure, was adopted differently in various settings over time. Diverse health and care providers benefited differently from the information held in the KIS, with varying expectations regarding what constituted useful information based on their professional roles. These expectations varied, shaped by users’ professional roles as well as information needs, and only became evident as the KIS was adopted across different settings. They revealed how various providers’ specific requirements and perspectives influenced their expectations and use of the KIS. These expectations evolved as the expectations of integrated care evolved and more significantly after national initiatives diverted the use of KIS to new missions, necessitating workarounds and repair work to extend the health care–centric information infrastructure to new settings and to realign the KIS with its intended purposes.

Formal workarounds were used to extend the health care–centric information infrastructure to social care settings and to ensure access to the KIS during system outages. These workarounds included creating proxy access for a few nonclinical professionals (eg, social workers from councils and social care providers) involved in MDTs to view the KIS using hospital-based information systems. This allowed social workers and carers to access the KIS in a digital format and communicate with GPs using the secure emailing network. Formal workarounds also involved the development and sharing of contingency plans by vendors, health boards, and organizations with users to address unexpected technical barriers that hindered access to the KIS.

While formal workarounds facilitated access for new users and the development and sharing of contingency plans, informal user-driven repair work was used to gather and share relevant information from alternative sources to update the KIS. These user-driven repair works were subject to the affordances of the users, that is, their role and consequent level of access to the existing information infrastructure, digital skills, willingness, subjective perceptions of integrated care, organizational practices, and leadership. Users leveraged various interconnected systems within the information infrastructure, such as local datasets, other EHRs, and care plans, to fulfill their information needs, even though these systems were originally designed for different purposes. Over time, these locally developed repair strategies became embedded within practice, influenced patterns of the KIS’s adoption across settings, and contributed to shaping the evolution of the regional information infrastructure.

### Integration With the Literature

This research aligns with existing literature, showing that information infrastructures evolve over long periods, gradually embedding new technologies within user groups and becoming shaped by established organizational practices, information needs, and existing information systems [3,4,7,21,27]. Previous evaluations of summary care records have highlighted challenges associated with information quality, interoperability, and aligning shared records with the information needs and workflows of different professional groups [1,3,5,7]. However, these evaluations have often examined summary care records at particular points in time, providing limited insight into how such technologies evolve as part of broader information infrastructures [1]. Our previous research examined how the KIS and associated infrastructures developed through successive waves of infrastructural growth [7]. This study extends that work by examining what happens after infrastructural expansion, focusing on how users domesticate, maintain, and transform established infrastructures through everyday practices of repair and workaround. Building on this perspective, this study illustrates how the KIS continued to evolve as it became embedded across diverse health and care settings, where different professional groups developed context-specific interpretations of its purpose, functionality, and information requirements. Jarrahi et al [27] further emphasize that information infrastructures are open and generative, developing through interaction with new settings where they combine with existing practices and information needs. By examining a digital technology used for collaborative work [5-7], this study illustrates how the KIS was translated across different settings over time and embedded within the installed base, highlighting the generative nature of the information infrastructure as it evolved to support health and social care integration. Across these settings, user groups differed in their understanding of the usability of the KIS, which changed over time, as they started to adopt the KIS. For instance, the KIS was designed to share information from GP records with other people involved in delivering care. However, as the KIS was implemented and adopted across settings over time, user groups developed differing interpretations of what constituted key information. OOH staff, for instance, benefited from having up-to-date future care plans recorded in the KIS, but this required GPs to collate information from various information sources to update the KIS. Consequently, their views on how the KIS should be updated and what it should contain varied according to the users’ professional roles and information needs, informing the repair work they used, such as referring to other EHRs and datasets to which they had access. Findings from the study illustrate the range of strategies organizations and users used to update information in the KIS, including formal workarounds to extend the KIS into new settings and informal, user-driven repair work to access and update with relevant information. These findings demonstrate that information infrastructures integrating health and care providers are open and evolve generatively over extended periods, with workarounds and user-driven repair practices shaping their extension and adoption across settings [3-7,27].

This study also aligns with the literature highlighting that workarounds are an integral part of technology implementation and adoption [10,11]. Several workarounds observed in this study are consistent with the literature describing formal, interim measures used to address limitations around technology usability [11]. Formal workarounds identified in the study included the development of contingency plans and the provision of interim user guides by vendors, offering temporary instructions to support users in navigating the technology during periods of limited system functionality. Contingency plans involved using alternative information systems and paper-based records as intermediaries to maintain continuity of care in the event of system failures. Finally, formal workarounds were also used to extend the health care–centric information infrastructure to nonclinical settings. These findings illustrate that workarounds are strategies used by individuals and organizations to navigate technological or procedural constraints, and that they play a critical role not only in technology implementation and adoption but also in the evolution of information infrastructures [8,10-12].

Information infrastructure and the repair work it entails have been examined from various perspectives. Scholars note that information infrastructures are prone to breakdown and require repair as part of their maintenance [28,29]. However, existing studies focus on visible and invisible repair activities, the use of material tools, and the power dynamics during infrastructural failure, but pay limited attention to repair work in the context of the long-term evolution of infrastructures for integrating health and care providers [28,29]. This study contributes to the literature by highlighting that the evolution of information infrastructures for integrating health and social care providers necessitates user-driven repair work for its maintenance. User-driven repair work involved drawing on local datasets and other EHRs—containing overlapping information but designed for different purposes—to identify and share relevant information for updating the KIS. These datasets and information present in different systems can be conceptualized as digital assemblages or personal information infrastructures, which users engaged with to support their information needs [30]. A digital assemblage is a combination of digital and physical resources that users interact with to achieve specific objectives [27,30,31]. By analyzing how users engaged with digital assemblages during infrastructural breakdowns to meet their information needs, this study shows that infrastructural growth is also driven by bottom-up strategies and enacted through user-driven repair work.

Finally, this research contributes to the theory of workarounds by further elucidating the relationships between environment, strategies, and infrastructure [10-12]. The theory of workarounds seeks to classify the different types of workarounds used by users, identify the conditions under which workarounds emerge, and explain how sociotechnical systems evolve through the continued use of workarounds [10]. Central to this theoretical perspective is the argument that the enactment of workarounds by users, organizations, developers, and implementers arises from the dynamic interplay between environment, strategies, and infrastructure. According to Alter [10], environment refers to the broader organizational, cultural, technical, and regulatory context in which a technology is implemented, whereas strategies denote organizational- and vendor-level approaches to technology development, implementation, and customization. While the theory of workarounds emphasizes the relationship among these elements, subsequent studies have largely focused on the interplay between environment and strategies [11,12], giving limited attention to infrastructure. This study demonstrates that access to the information infrastructure enabled users to undertake repair work aligned with their specific information needs, thereby generating new affordances. For instance, access to the information infrastructure allowed clinical users to draw on multiple information sources that contained information analogous to the KIS. Examples include hospital staff accessing other EHRs and local datasets to retrieve information related to multimorbidity, polypharmacy, and risk of hospital readmission, which were then used to inform their clinical decision-making needs. Similarly, access to the information infrastructure enabled nonclinical (social workers and social care workers) users to leverage the infrastructure to access the KIS and communicate relevant updates to GPs via the secure email system. These cases demonstrate how users used the information infrastructure to undertake diverse repair work across different settings, thereby creating affordances. In turn, this contributes to theorizing how users leverage the infrastructure to implement different types of workarounds and repair work within specific contexts, where organizations and vendors pursue distinct approaches to technology development, customization, and adoption.

### Strengths and Limitations

This study offers a methodological contribution by demonstrating the value of combining longitudinal and contextual analytical approaches with a multimethod qualitative case study design to examine the evolution of information infrastructures. The integration of the BoAP and TPOM frameworks enabled examination of both the historical trajectory of the KIS and the contextual factors shaping its adoption and use across different settings. While BoAP supported reconstruction of the KIS’s development and evolution over time, TPOM facilitated analysis of the technological, sociotechnical, organizational, and macroenvironmental conditions influencing its adoption in practice. By triangulating historical and contemporary documentary evidence with interviews and ethnographic observations, the study further strengthened this longitudinal perspective by reconstructing the development, adoption, and use of the KIS across multiple settings and time periods.

This approach enabled examination of how contextual factors, historical trajectories, organizational dynamics, and evolving understandings of integrated care shaped technology adoption over time, while capturing long-term trends that are difficult to observe through cross-sectional or short-term implementation studies [3-5]. Combining documentary evidence with participants’ retrospective accounts and observations of contemporary practice allowed different sources of evidence to be compared and contextualized, providing a richer understanding of how information infrastructures evolve through successive phases of development, implementation, domestication, and everyday use. This perspective made it possible to examine how challenges relating to usability, organizational practices, and macroenvironmental conditions changed over time and across settings, dynamics that are often overlooked in snapshot evaluations of digital technologies [19].

However, reconstructing longitudinal processes primarily through retrospective accounts and documentary evidence, rather than prospective longitudinal ethnography, constitutes a limitation. Although triangulation with observations strengthened the analysis, the study could not fully capture the granular, real-time negotiations, decision-making processes, and implementation activities that occurred during the early phases of KIS development and adoption. Additionally, while BoAP enabled examination of historical trajectories and TPOM supported contextual analysis across settings, the use of retrospective data meant that changes in workforce digital skills, organizational practices, and technological arrangements could not be systematically observed as they unfolded.

Adoption strategies mentioned in the study are specific to users in the Lothian region, and this contextual specificity also limits the generalizability of the findings. The various care pathways, health and care professionals involved, and technological arrangements may differ in other regions of Scotland. Studying adjacent contexts would enable comparative insights into how local contingencies shape both processes and outcomes and would also allow the findings to be situated more robustly within the broader context of integration. Finally, responsibilities for care provision and approaches to digital integration vary internationally, limiting the direct transferability of these insights to health system contexts beyond Scotland. However, the study offers valuable and transferable theoretical contributions: it demonstrates how the domestication of digital technologies evolved over time and highlights the ongoing repair work and workarounds required to maintain and extend information infrastructures.

### Implications for Policy and Practice

Adoption of digital technologies for integrating health and care providers is a dynamic process, shaped by broader macroenvironmental conditions, evolving user needs, and organizational practices. Consequently, policies aimed at supporting digital technologies in integrated care must incorporate mechanisms for continuous feedback and periodic evaluation, particularly following major changes relating to their use. Such iterative assessments enable implementers, policymakers, and vendors to identify emerging challenges, understand the strategies that users develop in practice, and adjust technologies or implementation approaches accordingly. Findings from the study highlight a range of user-driven repair practices undertaken to maintain the infrastructure. Identifying such local practices is useful, as it provides insight into shortcomings in design and can support further iterations of technology development and infrastructural growth, as well as the streamlining and regulation of these practices. These repair practices can also serve as indicators of implementation and adoption barriers, highlighting where additional resources, training, governance mechanisms, or changes to existing workflows may be required.

The findings suggest that continuous quality improvement should not focus solely on increasing user adoption, but also on identifying where repair work and workarounds emerge during routine use. These practices provide valuable indicators of mismatches between the intended use of digital technologies and evolving expectations of integrated care, organizational arrangements, and users’ information needs over time [7,32]. Rather than viewing repair practices simply as deviations from intended implementation, they can be used to identify challenges, reveal areas where technologies no longer align with everyday practice, and inform iterative improvements of digital infrastructures. Table 2 summarizes the key implementation and adoption challenges identified in this study, their impact on KIS use, the formal workarounds and user-driven repair practices that emerged in response, and the associated implications for policy and practice.

**Table 2:** Recommendations for policy and practice.

Implementation and adoption challenges	Impact on KIS^a^ use	Formal workaround or repair response	Recommendations for policy and practice
Variation in GP^b^ capacity and responsibility for maintaining records	Inconsistent updating and completeness of KIS information	Local prioritization approaches, MDT^c^ involvement, structured information sharing from community teams	Distribute responsibility for maintaining shared records across multidisciplinary teams, supported by dedicated time, organizational support, and clear governance arrangements.
Repurposing of KIS during COVID-19	Increased number of records with limited future care planning information	GP-led review and restoration of KIS information quality	Ensure that future policy changes align with the intended function of shared records and include plans for maintaining information quality during and after implementation.
Limited interoperability across health and social care systems	Reduced ability of social care providers to directly contribute information	Proxy access, secure email, templates, and intermediary roles	Strengthen interoperability across semantic, organizational, and legal levels by addressing existing barriers and learning from user-developed workarounds. Develop structured mechanisms for extending information infrastructures that enable social care professionals to contribute information while maintaining appropriate governance and accountability.
Differences in professional information needs and documentation practices	Variation in what information was considered relevant across settings	Negotiation between professional groups and development of local documentation processes	Engage different user groups when designing and revising shared records to understand their distinct roles, information needs, and workflows, ensuring that records support the diverse requirements of clinical and care settings.
Technical limitations and system outages	Temporary disruption to access and established workflows	Contingency plans, alternative systems, vendor-supported workarounds	Develop resilient contingency arrangements and involve users in testing workflow changes before implementation.

^a^KIS: Key Information Summary.

^b^GP: general practitioner.

^c^MDT: multidisciplinary team.

Importantly, improvement efforts should not seek to eliminate all workarounds, as some locally developed practices enabled users to overcome limitations within the existing infrastructure and supported continued use of the KIS in different settings. Instead, organizations should identify which workarounds represent valuable context-specific solutions and which indicate persistent system limitations requiring intervention. This requires balancing standardization of digital infrastructures with flexibility to accommodate differences in organizational contexts, professional roles, and local workflows.

Moreover, national and regional initiatives must aim to strengthen the digital competencies of health and care professionals and establish networks for knowledge exchange, which are crucial to sustaining adoption and use at different settings over time. These initiatives must recognize the heterogeneity of stakeholders: different organizations vary considerably in their digital maturity, resource availability, and leadership capacity. Therefore, policy interventions should avoid uniform approaches and instead provide long-term support tailored to organizational contexts. This includes ensuring that sufficient workforce capacity, training, and organizational support are available to sustain information-sharing practices over time. The analysis also demonstrates the importance of communities of practice and intermediary organizations in sharing locally developed strategies and supporting wider adoption across settings.

Findings from the study also illustrate how users relied on multiple digital assemblages to meet their information needs when existing infrastructures did not fully support their activities. Future adoption strategies should therefore anticipate how emerging sources of information, such as AI-supported documentation tools or patient-generated data from wearable devices, may interact with existing information infrastructures. Policies must consider how these sources can be incorporated while maintaining appropriate governance arrangements, interoperability, and alignment with the needs of diverse users.

### Conclusions

Using the KIS in Scotland as a case, this study illustrates how regional information infrastructures for integrating health and care providers evolved over time, embedding technologies within local routines, practices, and existing systems, while being shaped by professional roles and information needs. Adoption occurs incrementally, with formal workarounds extending health care–centric infrastructure to new settings and informal user-driven practices facilitating adaptation across diverse local contexts and supporting ongoing infrastructural maintenance. The study offers theoretical insights into how users domesticate digital technologies across diverse health and care settings over time and how infrastructural growth is facilitated through workarounds and user-driven repair work. Importantly, policymakers and technology vendors must recognize that technology deployment alone is insufficient; they need to account for evolving user needs and the diverse strategies users use to make technologies functional in practice. This requires continuous evaluation of implementation challenges, including resource constraints, interoperability limitations, and changing information needs, while supporting locally developed solutions that enable technologies to remain useful across diverse settings. Understanding these practices can inform how technologies become embedded within the infrastructure, identify and manage workarounds as well as user-driven strategies that support infrastructural growth, and guide future innovation.

## Data Availability

The datasets generated or analyzed during this study are available from the corresponding author on reasonable request.

## Appendix

Multimedia Appendix 1 Topic guide for interviews.

Multimedia Appendix 2 Protocol for observation.

Multimedia Appendix 3 Selection criteria for documents.

Multimedia Appendix 4 Codes and themes emerging from the data.

Multimedia Appendix 5 COREQ checklist.

## Abbreviations

BoAP: biography of artifacts and practices
COREQ: Consolidated Criteria for Reporting Qualitative Research
ECS: Emergency Care Summary
EHR: electronic health record
EHSCP: Edinburgh Health and Social Care Partnership
EMREC: Edinburgh Medical School Research Ethics Committee
GMS: General Medical Service
GP: general practitioner
KIS: Key Information Summary
MDT: multidisciplinary team
NHS: National Health Service
OOH: out-of-hour
QI: Quality Improvement
REDMAP: Ready, Expect, Diagnosis, Matters, Actions, and Plans
SAS: Scottish Ambulance Service
SCI: Scottish Care Information
SPARRA: Scottish Patients at Risk of Readmission and Admission
TPOM: technology-people-organization-macroenvironment
