# Supplementary material for: Adoption Strategies and Workarounds in the Evolution of Information Infrastructures Integrating Health and Social Care Providers in Scotland: Multimethod Qualitative Case Study of the Key Information Summary
Source: JMIR Med Inform. 2026 Oct 20;14:e94376. doi: 10.2196/94376 (PMC13732172; doi:10.2196/94376)
Supplement: Supplementary file 2 [file medinform_v14i1e94376_app1.docx]

**Topic Guides for interview**

Introduction and icebreaker-users/implementers

1. Could you share a bit about your role and involvement with KIS?
2. How long have you been involved in using KIS in this current organisation?

Understanding the roll out of the initiative/implementation – Users

1. What information systems were used prior to KIS?
2. When was the KIS introduced? And by whom?
3. What incentives did you or your organisation have to implement the KIS?
4. Were the objectives of the intervention clear?
5. Could you elaborate on how your experience was with implementation process?
6. What according to you were the facilitators and barriers that emerged during the implementation?
7. Were there other similar initiatives from where you could draw learnings during the roll-out?
8. Were there any other technologies/mechanisms to collect the information generated? If so, is the technology interoperable with other information systems in use?
9. Who were the initial participants and were they all homogenous in terms of digital maturity?
10. Did all users from the different settings have access to the information that is being generated and shared?

Understanding usability of the technology

1. Was the technology easy to use and was it according to your expectations? Could you share on your experience using KIS?
2. Did you receive any training on how to use KIS?
3. Could you elaborate on how you gained familiarity with KIS?
4. What elements of KIS worked well for you or what did not?
5. How has the usage of KIS helped you with your workload?
6. What has been your experience engaging with other users of KIS?
7. Has the use of KIS changed over time, from the time of implementation to now?
8. What use do you or your organisation have with the information generated and shared?
9. What information do you think needs to be incorporated to into KIS to meet its purpose?
10. Has the use of KIS changed over time, from the time of implementation to now?

Understanding adoption of the technology -

1. Was KIS adaptable to your work setting?
2. Was KIS apt for your job role?
3. What were the challenges in adopting KIS to your daily routine/ work practice?
4. Has the technology changed any existing work practices?
5. What were your experiences from communicating with other service providers using KIS/Connect care?
6. Were there mechanisms to share learnings across other user groups?
7. What mechanisms were in place to help support the adoption of KIS?
8. What organisational mechanisms were in place to share and retain knowledge?
9. Are there any other interventions/policies that could change the course of adoption of KIS?
10. How has the adoption of KIS been for you in the light of new policy demands and technology upgrades?

Key lines of enquiry specific to Implementers

1. Could you elaborate on your role as an implementer?
2. Who were the initial stakeholders during the implementation of KIS?
3. What were the expectations of implementing KIS?
4. What were the incentives for implementers and users to implement KIS?
5. According to you, what were the experiences emerging from its use? Did the expectations match their needs as well? Was it similar across all the participating entities?
6. What do you think has changed from the time the technology was implemented?
7. How has the policy landscape changed from the time of implementation of KIS
8. How has the expectations changed over time?
9. What measures are taken to meet these new expectations?
10. What mechanisms were in place or are required for successful implementation of KIs across new services?
11. How do you think this technology will be sustainable in the future?

Key lines of enquiry specific to vendors/ suppliers

1. How did you get involved with KIS?
2. What were the initial expectations around developing and implementing KIS?
3. Could you elaborate on how your experience was with development and implementation process?
4. What according to you were the facilitators and barriers that emerged during the initial stages of design and roll out?
5. Were there other similar initiatives from where you could draw learnings during the roll-out?
6. Were there any other technologies/mechanisms to collect the information generated? If so, is the technology interoperable with other information systems in use?
7. How has the supplier landscape changed from the time of implementation of KIS?
8. What mechanisms are in place to support the enrolment and implementation of new KIS across new services?
9. How do you think this technology will be sustainable in the future?
10. What do you think has worked well for KIS? And what could be done better?
