# Supplementary material for: Adoption Strategies and Workarounds in the Evolution of Information Infrastructures Integrating Health and Social Care Providers in Scotland: Multimethod Qualitative Case Study of the Key Information Summary
Source: JMIR Med Inform. 2026 Oct 20;14:e94376. doi: 10.2196/94376 (PMC13732172; doi:10.2196/94376)
Supplement: Supplementary file 3 [file medinform_v14i1e94376_app2.docx]

**Protocol for Observation**

**Purpose of the meeting/event**

- What is the objective of the event or meeting, and what resources are provided to contextualize or support the information discussed?
- Who is responsible for organizing the event, and how is the KIS referenced within this context?

**Brief description of the various actors involved and their roles**

- Affiliation and job title of the actors involved, as well as the nature of their involvement/role in the context of the meeting.

**Insights** **into how the KIS was used and adopted in practice**

- What references are made regarding the use and adoption of the KIS, and which actors are making these references?
- What resources are being used to share learning across participating organizations and how is it shared? What resources are employed to facilitate learning across participating organizations, and through what mechanisms is this learning shared?
- What plans are being developed regarding the future use/adoption of the KIS?

**Insights into impacts – practice, user skills, behavior/attitudes, routine, and workflow**

- How are participating actors and organizations referring to the use and adoption of the KIS during the meeting?
- What issues are being reported and addressed?
- Which issues are not being reported or addressed, and at what level?
- What information sources or documents are being shared to participants?
- To what extent is there constructive rapport among participants, and between which groups?

**Reaction/response of actors to questions**

**Feelings of the researcher – researcher’s impressions/feelings in relation to the event/meeting observed**
