# Supplementary material for: Adoption Strategies and Workarounds in the Evolution of Information Infrastructures Integrating Health and Social Care Providers in Scotland: Multimethod Qualitative Case Study of the Key Information Summary
Source: JMIR Med Inform. 2026 Oct 20;14:e94376. doi: 10.2196/94376 (PMC13732172; doi:10.2196/94376)
Supplement: Supplementary file 4 [file medinform_v14i1e94376_app3.docx]

Main selection criteria for documents included the following:

- Documents providing context on the development, implementation, and use of the KIS in Scotland and the Lothian region.
- Documents relating to the integration of health and care services in Scotland
- Documents describing the functionalities and intended uses of the KIS.
- Documents relating to the development of the health and social care information systems used to access the KIS.
- Documents providing context on the implementation strategy and anticipated benefits of the KIS at both local and national levels.
- Documents providing information on the organizational context surrounding the implementation and use of the KIS.
- Documents documenting the history, progress, and evolution of the KIS implementation.
- Documents providing insights into the perspectives of stakeholder groups that were difficult to access through other data collection methods.
- Documents identifying lessons learned, anticipated challenges, and experiences relating to the implementation and use of the KIS from diverse stakeholder perspectives.
