# Supplementary material for: Adoption Strategies and Workarounds in the Evolution of Information Infrastructures Integrating Health and Social Care Providers in Scotland: Multimethod Qualitative Case Study of the Key Information Summary
Source: JMIR Med Inform. 2026 Oct 20;14:e94376. doi: 10.2196/94376 (PMC13732172; doi:10.2196/94376)
Supplement: Supplementary file 5 [file medinform_v14i1e94376_app4.docx]

# Codes and themes emerging from the data

| Abductive codes | | Second-order themes | Aggregate themes |
| --- | --- | --- | --- |
| **Socio-technical and socio organizational factors effecting use of KIS** | Demography and nature of interprofessional working | Shifts in GP service provision and data control | Intended use of the KIS and the information required for continuity of care |
|  | Different spaces and providers of care and the different information needs |  |  |
|  | GPs as custodians of data |  |  |
|  | Complex care needs and information requirements |  |  |
|  | Key information in KIS |  | Tensions between controllers and users of information |
|  | Different spaces and providers of care and the different information systems in use |  |  |
|  | Resource allocation in different regions and sectors |  |  |
|  | GPs having to update information in KIS from other sources |  |  |
|  | Diverse providers and workforce issues |  |  |
|  | Changes post-GMS contracts |  |  |
|  | Incentives for GPs and GP practices to update KIS |  |  |
|  | Personal incentives and motives to work |  |  |
| **Macroenvironmental and socio-organizational factors influencing the use of the KIS** | Top-down approaches during COVID-19 to identify vulnerable groups | Impact of top-down policies on information quality |  |
|  | Increased adoption of KIS during COVID-19 |  |  |
|  | Regional attempts in linking healthcare providers and local councils using the KIS pre-COVID |  |  |
|  | Reduced information quality with KIS stating “created as a part of COVID-19 protocol” |  |  |
|  | Increased workload to identify useful KIS |  |  |
|  | Information desired for future care planning |  |  |
|  | Expectations of users (GPs) in using the KIS for Future care planning |  |  |
|  | Expectations of other clinical users in using KIS for future care planning |  |  |
|  | | | |
| **Technological, sociotechnical, and socio-organizational factors affecting the use of KIS** | Workarounds to extend the KIS to clinical settings | Formal workarounds to extend the information infrastructure | Formal workarounds to access and use the KIS |
|  | Workarounds to extend the KIS to non-clinical settings |  |  |
|  | Risk and trust in information sharing practices |  |  |
|  | Training care providers in using the KIS by health and social care partnerships |  |  |
|  | Existing forms for FCP |  |  |
|  | materiality of various forms of information sources |  |  |
|  | System outage and ransomware attack in OOH settings |  |  |
|  | Legitimization of workarounds |  |  |
|  | Increased workload due to technology outage | Formal workarounds to access the information infrastructure during system outages |  |
|  | workarounds and contingency plans |  |  |
|  | Technology outage in GP settings |  |  |
|  | Technology outage in hospital settings |  |  |
|  | Technology outage in social care settings and IT systems |  |  |
|  | Interruption to near-real-time and real-time data collection |  |  |
|  | Vendors recognizing workarounds |  |  |
|  | Version changes incorporating usability challenges |  |  |
|  | Vendors sharing workarounds |  |  |
|  | | | |
| **Technological, sociotechnical, and socio-organizational factors affecting the implementation and adoption of KIS in clinical and non-clinical settings** | Repair and maintenance in secondary care settings to retrieve up-to-date information | User-driven repair work in clinical settings | Repair work to adopt and use the KIS |
|  | Proxy data sources - datasets |  |  |
|  | Proxy data sources – algorithmic models |  |  |
|  | Multimorbidity as a proxy source to identify vulnerable groups needing KIS |  |  |
|  | Frailty as a proxy source to identify vulnerable groups needing KIS |  |  |
|  | Repair work in certain GP practices |  |  |
|  | Acknowledging good practice |  |  |
|  | Digital skills of the user |  |  |
|  | Perceptions of user regarding digital technologies for care |  |  |
|  | Volunteering to keep the information up-to-date |  |  |
|  | Sharing good practice |  |  |
|  | Role of leadership in sustaining good practice in clinical settings |  |  |
|  | Communities of practice sharing and legitimizing user-driven practices at scale |  |  |
|  | Non-digital proxy sources in care settings | Maintenance and repair work in nonclinical settings |  |
|  | Epistemic practices in record-keeping |  |  |
|  | Multiple material forms of KIS and problems of near real-time data |  |  |
|  | Digital transformation of social care providers |  |  |
|  | Human nodes in information sharing |  |  |
|  | Information desired from different pathways - dementia |  |  |
|  | Information desired from different pathways – long term care |  |  |
|  | Information desired from different pathways - reablement |  |  |
|  | Volatile workforce and knowledge retention |  |  |
|  | Role of leadership in sustaining good practice in non-clinical settings |  |  |
|  | Recognising a person's choice as dynamic and subject to change |  |  |
|  | Subjective notions of digital technologies for care |  |  |
